# Abbreviated non-contrast magnetic resonance enterography for Crohn’s disease: a reliability study

**DOI:** 10.1007/s11547-026-02227-8

**Published:** 2026-06-05

**Authors:** Valentina Caliendo, Vittorio Patanè, Luca Marinelli, Anna Russo, Maria Chiara Brunese, Francesco Selvaggi, Antonietta Gerarda Gravina, Salvatore Cappabianca, Alfonso Reginelli

**Affiliations:** 1 Department of Diagnostic Imaging, AORN “S.Anna e S.Sebastiano”, Caserta, Italy; 2 Department of Precision Medicine, University of Campania “Luigi Vanvitelli”, Piazza Luigi Miraglia 2, 80138 Naples, Italy; 3 Department of Advanced Medical and Surgical Sciences, Università Degli Studi Della Campania “Luigi Vanvitelli”,, Naples, Italy

**Keywords:** Magnetic resonance enterography, Crohn’s disease, Inflammatory bowel disease, Non-contrast magnetic resonance imaging, Abbreviated protocol, Diagnostic accuracy

## Abstract

**Purpose:**

To evaluate the diagnostic reliability and time efficiency of an abbreviated *non-contrast* magnetic resonance enterography (MRE) protocol for assessing Crohn’s disease activity.

**Methods:**

This retrospective study included 312 patients who underwent MRE between March 2024 and November 2025. An abbreviated dataset was derived from each full examination by masking sequences not included in the abbreviated protocol, such as all contrast-enhanced acquisitions. Two radiologists independently reviewed the abbreviated and full datasets in separate sessions, fully blinded to clinical information and the alternative dataset. The interpretation of the full MRE dataset served as the reference standard. Outcomes included agreement (Cohen’s kappa), sensitivity and specificity for active disease, image quality ratings (4-point scale), and estimated acquisition time.

**Results:**

Agreement for overall disease activity was substantial (*κ* = 0.83), with sensitivity of 92.3% and specificity of 94.6% for detecting active disease. Agreement was strong for bowel wall thickening (*κ* = 0.85), mural edema (*κ* = 0.81), and the comb sign (*κ* = 0.79). Estimated acquisition time was 44% shorter for the abbreviated protocol (21.6 vs. 38.4 min), while diagnostic image quality was preserved (mean 3.5 ± 0.6).

**Conclusion:**

An abbreviated non-contrast MRE protocol provides reliable assessment of Crohn’s disease activity with markedly reduced scan time and maintained image quality, supporting its use in selected follow-up scenarios.

## Introduction

Crohn’s disease is a chronic inflammatory bowel disorder marked by segmental, transmural inflammation that can affect any part of the gastrointestinal tract, most notably the terminal ileum and proximal colon [1–6]. Its relapsing–remitting nature and tendency to emerge in early life create a significant long-term burden, frequently requiring ongoing monitoring of inflammatory activity and associated complications. [7–10].

There is no single diagnostic gold standard for Crohn’s disease; diagnosis and monitoring rely on the integration of clinical, laboratory, endoscopic, and imaging findings [11–17]. Ileocolonoscopy remains central for evaluating mucosal disease and obtaining histologic samples, but it is limited in its ability to assess transmural inflammation, stenoses, and extramural complications such as fistulas and abscesses [18–22]. Cross-sectional imaging, therefore, plays a pivotal role in treatment planning and follow-up, particularly when symptoms, biomarkers, and endoscopic findings do not fully capture the extent of transmural disease [18, 23–27].

Magnetic resonance enterography (MRE) has become a mainstay in Crohn’s disease imaging because it provides excellent soft-tissue contrast without ionizing radiation and enables evaluation of both mural and extramural disease [18, 28–31]. However, standard MRE protocols are often time-consuming and frequently include intravenous gadolinium-based contrast administration, which may reduce their feasibility in repeated follow-up examinations [19, 20, 32–34]. These factors are especially relevant for patient groups requiring lifelong monitoring and for high-volume centers where scanner time is a limited resource [35–37].

In this context, abbreviated non-contrast MRE approaches combining high-resolution T2-weighted imaging and diffusion-weighted imaging have attracted increasing interest. Such strategies aim to reduce the need for contrast administration while preserving diagnostic information on inflammatory activity and complications [38–41]. The purpose of this study was to evaluate the diagnostic performance of an abbreviated non-contrast MRE protocol and its agreement with the standard full protocol in a large cohort of patients with suspected or established Crohn’s disease. We hypothesized that the abbreviated approach would show high agreement and comparable diagnostic performance in assessing clinically meaningful disease activity, supporting its use for follow-up imaging in selected clinical scenarios.

## Materials and methods

### Study design, setting, and ethics

This retrospective observational study was conducted at the (anonymized data due to blind peer review). The study was performed in accordance with the Declaration of Helsinki and was approved by the Ethics Review Committees of the (anonymized data due to blind peer review) (Protocol No. 145786/i/2022). Ethical approval was obtained prior to data retrieval and analysis. The requirement for informed consent was waived due to the retrospective design and the use of fully anonymized data.

### Patient population

**Fig. 1:**
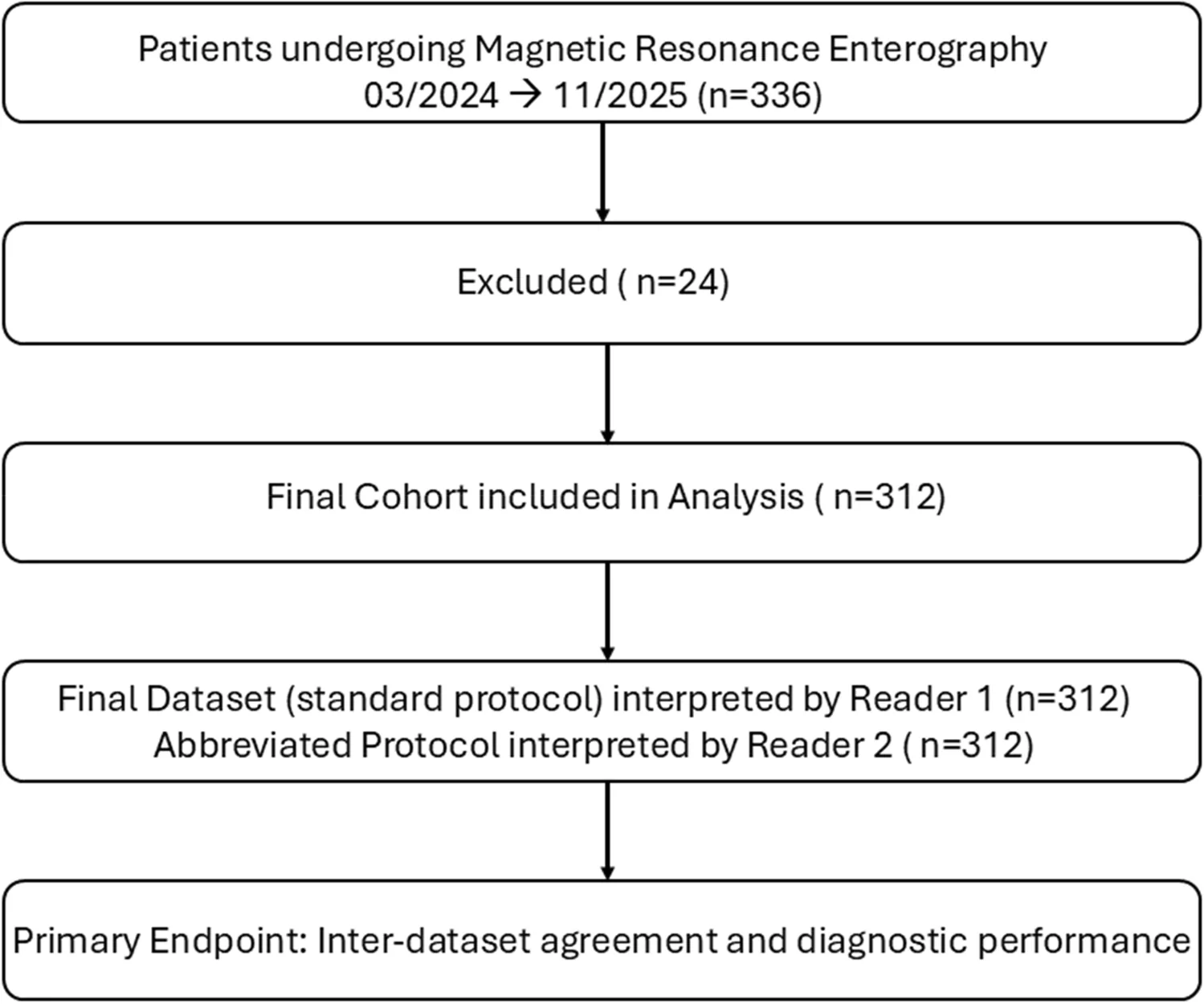
Flow diagram of patient selection, exclusions, and dataset definition. The abbreviated non-contrast dataset was derived from the full examination by masking sequences not included in the abbreviated protocol, such as all contrast-enhanced acquisitions

Consecutive patients with suspected or established Crohn’s disease who underwent MRE between March 2024 and November 2025 were screened. Overall, 336 examinations were initially identified. Patients were excluded due to non-diagnostic image quality precluding interpretation (*n* = 8), contraindication to gadolinium-based contrast agents due to renal insufficiency (*n* = 12), and other reasons (*n* = 4). The final cohort comprised 312 patients. A patient selection flow diagram is provided in Fig. 1.

### Patient preparation and antiperistaltic protocol

All patients fasted for at least 4 h prior to MRE. To achieve adequate bowel distension, patients ingested polyethylene glycol (PEG) diluted in water, with the administered volume adjusted according to height and weight. Pediatric patients and individuals with prior intestinal resection received a reduced volume tailored to body weight and tolerance. To reduce motion artifacts related to peristalsis, hyoscine butylbromide was administered intravenously at a total dose of 40 mg, split into two 20 mg doses, when not contraindicated. The first dose was given before balanced steady-state free precession imaging, and the second dose was given before unenhanced T1-weighted acquisitions. All examinations were carried out according to latest guidelines [42–44].

### Magnetic resonance enterography acquisition and dataset definition

All examinations were performed on a 1.5-T MRI system (SIGNA Voyager, GE Healthcare, USA) using a 16-channel phased-array body coil, with patients scanned in the prone position.

The full MRE protocol included multiplanar localizers, T2-weighted single-shot fast spin-echo acquisitions (including a breath-hold 3D coronal sequence and additional coronal and axial acquisitions with and without fat suppression), balanced steady-state free precession sequences, and diffusion-weighted imaging (DWI) acquired with b-values of 0, 400, and 800 s/mm^2^, along with apparent diffusion coefficient (ADC) maps. Intravenous gadolinium-based contrast was administered for contrast-enhanced T1-weighted 3D gradient-echo imaging (LAVA), including multiphasic perfusion and an optional delayed phase acquired approximately 7 min after injection.

**Fig. 2:**
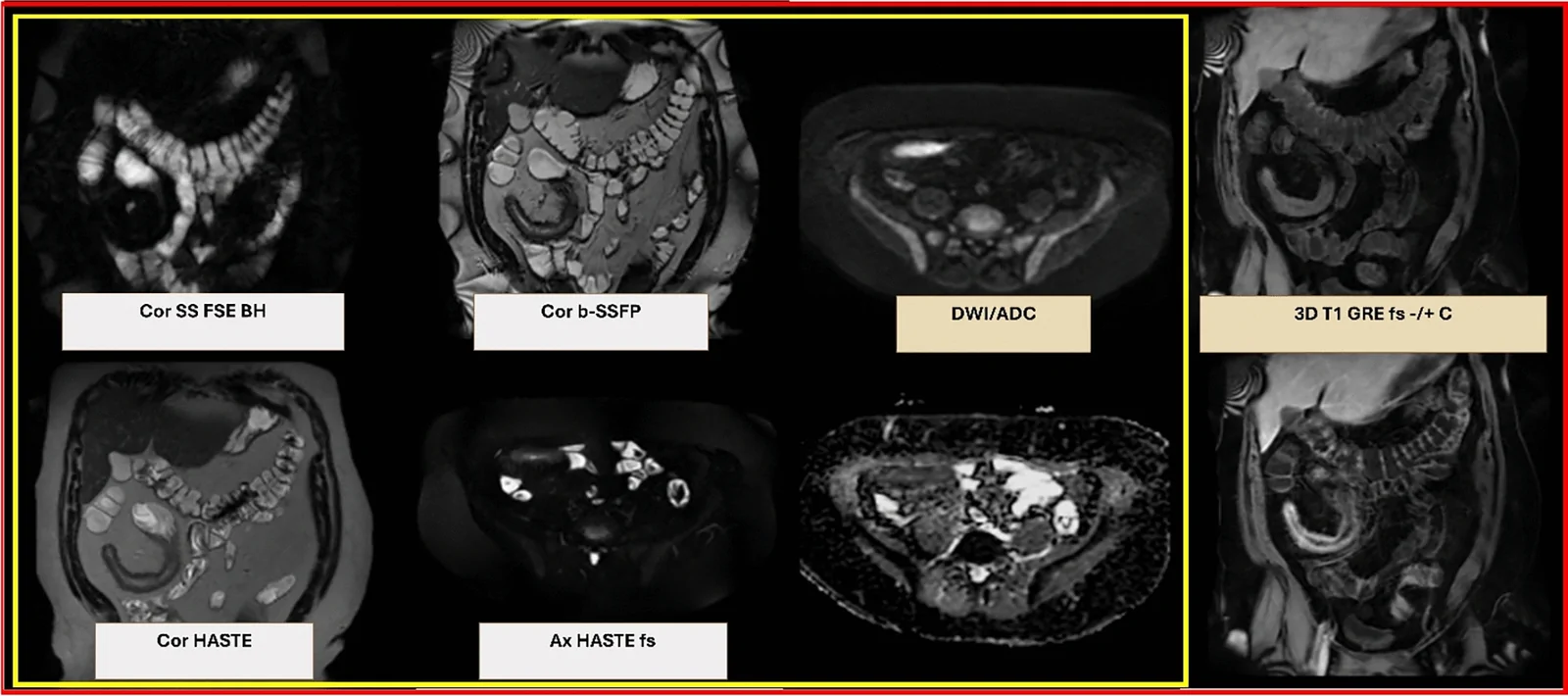
Patient-level overall activity classification by dataset. Counts of active and inactive Crohn’s disease classifications are shown for the full dataset and the abbreviated non-contrast dataset

The abbreviated non-contrast protocol was not acquired as a separate examination. Instead, an abbreviated non-contrast dataset was derived from the full examination by masking all sequences not included in the abbreviated protocol, such as all contrast-enhanced acquisitions. The abbreviated dataset consisted exclusively of T2-weighted imaging and DWI with ADC maps. The sequence composition is summarized in Table 1 and visually illustrated in Fig. 2.

**Table 1:** Sequence composition of the full MRE protocol and the abbreviated non-contrast dataset

Acquisition	Full protocol	Abbreviated protocol
Coronal 3D T2-weighted single-shot fast spin echo (breath-hold)	Included	Included
Coronal T2-weighted single-shot fast spin echo	Included	Included
Axial T2-weighted single-shot fast spin echo (with fat suppression)	Included	Included
Additional T2-weighted single-shot fast spin echo (with/without fat suppression, other planes)	Included	Included
Balanced steady-state free precession (e.g., FIESTA)	Included	Included
Diffusion-weighted imaging (*b* = 0, 400, 800 s/mm^2^) + apparent diffusion coefficient maps	Included	Included
Contrast-enhanced T1-weighted 3D gradient echo (e.g., LAVA), multiphasic and delayed phase	Included	Not-Included

The abbreviated dataset included T2-weighted imaging and diffusion-weighted imaging with apparent diffusion coefficient maps only; all contrast-enhanced acquisitions were excluded

### Image analysis

Two abdominal radiologists independently interpreted the imaging datasets under fully blinded conditions. Reader 1 (V.C. with 7 years of gastrointestinal radiology experience) interpreted the full dataset, while Reader 2 (A. R., with 15 years of experience) interpreted the abbreviated non-contrast dataset. Both readers were blinded to clinical history, laboratory values, and endoscopic results and had no access to the alternative dataset or the other reader’s assessment. The abbreviated dataset was presented as a standalone examination.

This reading strategy was selected to minimize recall bias that may occur when the same reader interprets two highly overlapping datasets derived from the same examination, and to approximate a pragmatic workflow in which abbreviated and standard protocols are interpreted independently. Accordingly, the primary agreement analyses were designed to quantify the inter-dataset agreement between the abbreviated and full datasets, as interpreted by two independent, blinded readers.

For each examination, the readers recorded predefined imaging features, including bowel wall thickening (graded by severity), segmental extent of involvement, mural edema, ulcers, perimural fluid, lymphadenopathy, the comb sign, restricted diffusion, and complications such as abscesses, fistulas, and strictures. Findings were recorded at the segment level and then summarized at the patient level.

Disease activity was evaluated using a two-tier approach. First, at the segment level, individual inflammatory features were recorded. Second, an operational definition of “active inflammation” was applied to provide a clinically meaningful patient-level classification. Mural edema was defined as mural hyperintensity on T2-weighted images, particularly when evident on fat-suppressed acquisitions. Restricted diffusion was defined as an increased signal on high-b-value DWI with a corresponding low signal on ADC maps relative to the adjacent bowel or reference segments. Ulcers were defined as discrete mucosal defects or deep mucosal depressions identified on high-resolution T2-weighted imaging and supported, when present, by focal restricted diffusion [45]. The comb sign and perienteric fluid were considered supportive signs of active transmural inflammation [46]. An “active segment” was defined as bowel wall thickening in association with at least one objective marker of inflammatory activity, including mural edema, ulcers, restricted diffusion, or clear perienteric inflammatory changes such as the comb sign or perienteric fluid [47]. At the patient level, “overall active disease” was defined as the presence of at least one active segment. In parallel, a broader descriptive category of “any inflammation-related abnormality” was used to capture the presence of at least one inflammatory imaging feature in any segment, irrespective of whether the composite definition of an active segment was met. Complications were defined using standard radiologic criteria. A stricture was recorded when luminal narrowing was associated with upstream dilation and/or clear mural thickening [48]. A fistula was recorded when an abnormal tract connecting bowel loops or bowel to adjacent organs was visualized [49]. An abscess was recorded when a fluid collection with a definable wall and surrounding inflammatory changes was identified, with restricted diffusion used as a supportive sign, when present [50].

### Image quality and acquisition time

Overall image quality was rated on a 4-point scale (1 = poor, 2 = fair, 3 = good, 4 = excellent), considering bowel distension, motion artifacts, and signal-to-noise ratio. The acquisition time for the full protocol was obtained from scanner time stamps. Because the abbreviated protocol was derived from the full examination and not acquired separately, acquisition time for the abbreviated dataset was estimated by summing the acquisition times of the sequences included in the abbreviated protocol.

### Quantitative assessment and clinical correlation

Inflammatory activity was quantified using the simplified Magnetic Resonance Index of Activity (sMaRIA) [51], computed from variables available within each dataset to allow direct comparison between full and abbreviated readings. When available, imaging findings were correlated with C-reactive protein, fecal calprotectin, and ileocolonoscopy performed within 30 days of MRE. Analyses involving biomarkers or ileocolonoscopy were restricted to patients with available data, and missing values were handled by case-wise exclusion for the corresponding analyses.

### Reproducibility assessment

To evaluate intra-observer reproducibility, a subset of 30 examinations was re-read by the same radiologists after a 4-week interval under the same blinding conditions.

### Statistical analysis

Continuous variables were summarized as mean ± standard deviations, and categorical variables as counts and percentages. Agreement was assessed using Cohen’s kappa statistics, using weighted or unweighted approaches as appropriate, and reported with 95% confidence intervals (CIs). For patients with multiple affected bowel segments, a multilevel kappa approach was applied. Agreement analyses were primarily intended to quantify inter-dataset agreement between the abbreviated and full datasets as interpreted by two independent blinded readers, rather than interobserver agreement for the same dataset. The diagnostic performance of the abbreviated dataset for identifying patient-level overall active disease was calculated using the full dataset interpretation as the reference standard, reporting sensitivity, specificity, and predictive values. Correlation analyses with biomarkers were performed using appropriate correlation coefficients, and comparisons between datasets were carried out with tests selected according to data type and distribution. A two-sided *p* value < 0.05 was considered statistically significant.

## Results

### Study cohort and baseline characteristics

A total of 312 patients were included in the final analysis (164 females and 148 males; mean age, 38.5 years). The cohort included 72 pediatric patients (< 18 years) and 42 patients (13.5%) with a history of intestinal resection.

The terminal ileum was the most frequently involved region (62%), followed by the ascending colon (19%). Baseline characteristics and disease distribution are summarized in Table 2.

**Table 2:** Baseline demographic and clinical characteristics of the study cohort and disease distribution

Characteristic	Value
Patients, *n*	312
Age, mean (years)	38.5
Female sex, *n* (%)	164 (52.6)
Male sex, *n* (%)	148 (47.4)
Pediatric patients (< 18 years), *n* (%)	72 (23.1)
Previous intestinal resection, *n* (%)	42 (13.5)
Most frequently involved region:	Terminal ileum, 62%

### Disease activity classification and inter-dataset agreement

**Fig. 3:**
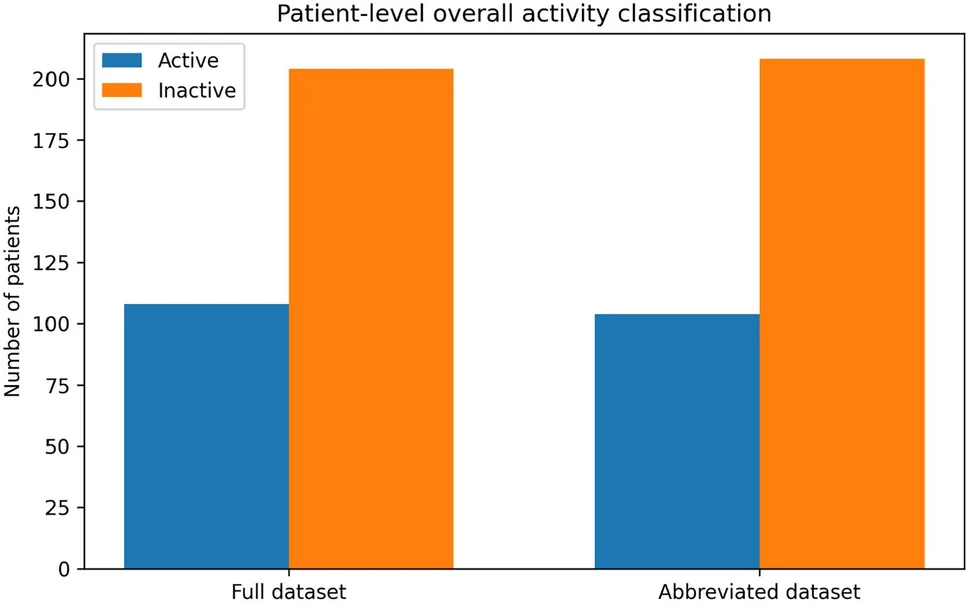
Overall image quality scores for the full and abbreviated datasets (4-point scale). Error bars indicate standard deviation

Using the descriptive category of any inflammatory-related abnormality, at least one inflammatory imaging feature in any bowel segment was identified in 176 patients (56.4%), whereas 136 patients (43.6%) showed no inflammatory imaging features. When applying the predefined composite criterion for patient-level overall active disease, the full dataset classified 108 patients (34.6%) as active and 204 as inactive, while the abbreviated dataset classified 104 patients (33.3%) as active and 208 as inactive. Agreement between the abbreviated and full datasets for patient-level overall activity was substantial (Cohen’s kappa = 0.83; 95% confidence interval, 0.78–0.88). Patient-level activity classification and diagnostic performance are summarized in Table 3 and illustrated in Fig. 3.

**Table 3:** Patient-level disease activity classification and diagnostic performance of the abbreviated non-contrast dataset using the full dataset interpretation as the reference standard

Outcome	Full protocol	Abbreviated protocol
Any inflammatory-related abnormality (at least one feature in any segment), *n* (%)	176 (56.4)	176 (56.4)
No inflammatory imaging features, *n* (%)	136 (43.6)	136 (43.6)
Overall active disease (patient-level composite), n (%)	108 (34.6)	104 (33.3)
Overall inactive disease, n (%)	204 (65.4)	208 (66.7)
Inter-dataset agreement for overall activity (Cohen’s kappa, 95% CI)	0.83 (0.78–0.88)	
Sensitivity for overall active disease (%)	92.3	
Specificity for overall active disease (%)	94.6	
Positive predictive value (%)	91.8	
Negative predictive value (%)		
	95.1	

Confidence interval (CI) refers to the kappa estimate

### Agreement on individual imaging features and complications

Inter-dataset agreement was strong for key inflammatory markers, including bowel wall thickening (kappa = 0.85), mural edema (kappa = 0.81), perienteric fluid (kappa = 0.81), and the comb sign (kappa = 0.79). Ulcer detection showed moderate agreement (kappa = 0.65). For complications, agreement ranged from moderate to substantial for fistulas, strictures, and abscesses (kappa range, 0.68–0.76). A clinically relevant nuance was the slightly reduced conspicuity of very small abscesses (< 2 cm) on the abbreviated dataset. Agreement metrics are summarized in Table 4.

**Table 4:** Inter-dataset agreement for key imaging features and complications

Imaging feature/complication	Cohen’s kappa
Bowel wall thickening	0.85
Mural edema	0.81
Comb sign	0.79
Perienteric fluid	0.81
Ulcers	0.65
Complications (fistulas, strictures, abscesses)	0.68–0.76

Values are reported as Cohen’s kappa

### Image quality and acquisition time

**Fig. 4:**
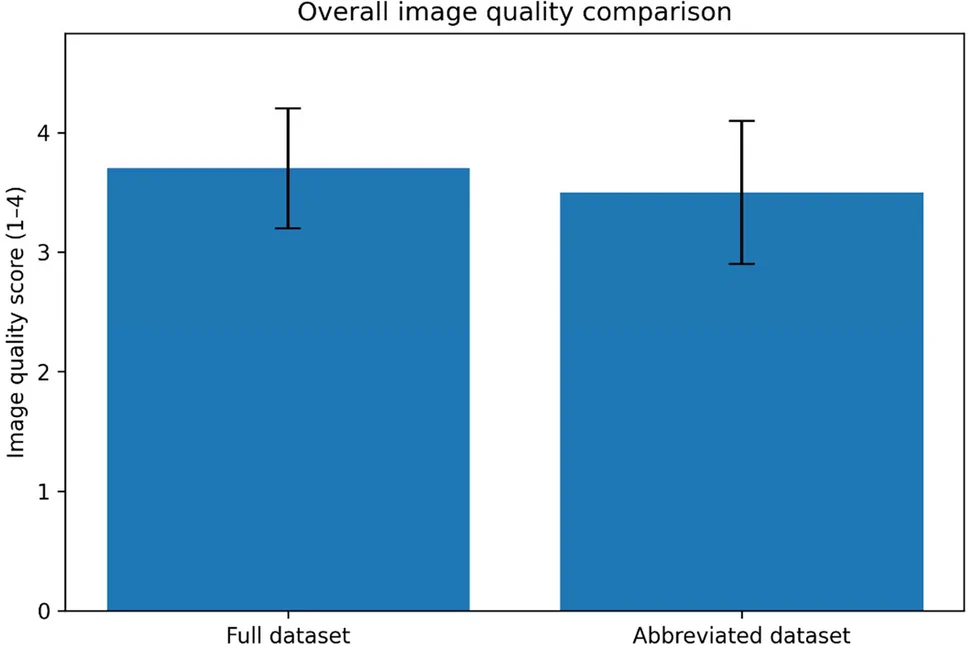
Acquisition time comparison between the full protocol and the abbreviated protocol (estimated). Error bars indicate standard deviation

**Fig. 5:**
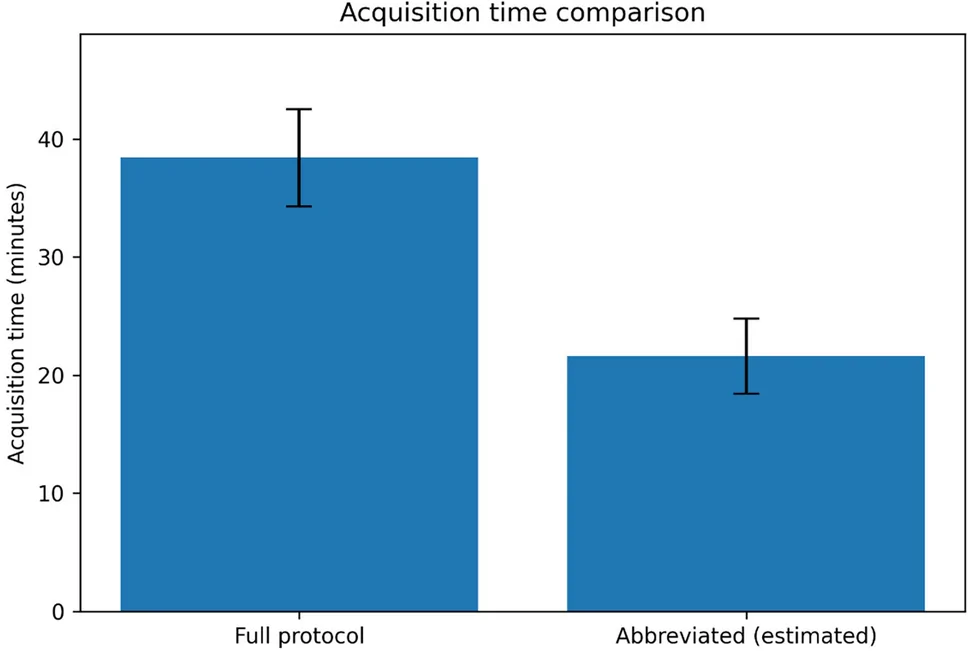
Acquisition time comparison between the full protocol and the abbreviated protocol. Error bars indicate standard deviation

Overall image quality was high for both datasets. Mean image quality scores were 3.7 ± 0.5 for the full dataset and 3.5 ± 0.6 for the abbreviated dataset on the 4-point scale, indicating a small reduction in perceived quality that remained diagnostically acceptable. Mean full-protocol acquisition time was 38.4 ± 4.1 min, whereas the estimated acquisition time for the abbreviated dataset was 21.6 ± 3.2 min, corresponding to an average reduction of approximately 44%. These comparisons are summarized in Table 5 and illustrated in Figs. 4 and 5.

**Table 5:** Comparison of overall image quality and acquisition time between the full dataset and the abbreviated dataset

Metric	Full protocol	Abbreviated protocol
Image quality score (4-point scale), mean ± SD	3.7 ± 0.5	3.5 ± 0.6
Acquisition time (minutes), mean ± SD	38.4 ± 4.1	21.6 ± 3.2 (estimated)
Time reduction (%)		44

Acquisition time for the abbreviated dataset is estimated

### Quantitative activity assessment and correlation with biomarkers and endoscopy

Simplified Magnetic Resonance Index of Activity values showed no statistically significant difference between datasets (*p* = 0.09). Correlations with inflammatory biomarkers were similar for both datasets, with correlation coefficients of 0.73 for the full dataset and 0.71 for the abbreviated dataset for C-reactive protein and fecal calprotectin. Among patients with ileocolonoscopy performed within 30 days (*n* = 86), agreement between imaging-based and endoscopic activity classification was high for both datasets, with kappa values of 0.79 for the full dataset and 0.76 for the abbreviated dataset (Table 6).

**Table 6:** Quantitative activity assessment and clinical correlations

Outcome	Full protocol	Abbreviated protocol
sMaRIA comparison (*p* value)	*p* = 0.09	*p* = 0.09
Correlation with biomarkers (C-reactive protein and fecal calprotectin), *r*	0.73	0.71
Agreement with ileocolonoscopy within 30 days (*n* = 86), Cohen’s kappa	0.79	0.76

sMaRIA indicates simplified Magnetic Resonance Index of Activity

### Reproducibility

Intra-observer reproducibility was evaluated in a subset of 30 examinations re-read after a 4-week interval. Intra-observer agreement was excellent for both datasets, with kappa values of 0.88 for the full dataset and 0.84 for the abbreviated dataset, indicating stable interpretation over time (Table 7).

**Table 7:** Intra-observer reproducibility assessed in a subset re-read after a 4-week interval under the same blinding conditions

Reproducibility analysis	Value
Subset size, *n*	30
Washout interval	4 weeks
Intra-observer agreement (Cohen’s kappa), full protocol	0.88
Intra-observer agreement (Cohen’s kappa), abbreviated dataset	0.84

## Discussion

This study demonstrates that an abbreviated non-contrast MRE protocol can provide a clinically reliable assessment of Crohn’s disease activity, with substantial agreement with the full protocol one for patient-level overall activity and strong concordance for the most actionable imaging markers of inflammation. In a large cohort that included pediatric patients and postoperative cases, the abbreviated dataset preserved diagnostic interpretability while offering a marked reduction in acquisition time and avoiding contrast-enhanced acquisitions. These findings support a practical role for contrast-free abbreviated MRE in follow-up imaging pathways, where repeated examinations are common and operational efficiency and patient burden are central considerations [52, 53].

Accurate evaluation of transmural inflammation and extramural complications is essential for treatment decisions in Crohn’s disease [33, 54–56]. Endoscopy remains indispensable for mucosal assessment and histology, but it provides only a partial view of disease biology and cannot reliably characterize transmural inflammation, proximal small-bowel involvement beyond reach, or extraintestinal disease manifestations [57–61]. Cross-sectional imaging fills this gap and has become a cornerstone in contemporary management strategies [62–64]. Within this context, magnetic resonance enterography is widely used because it avoids ionizing radiation and can simultaneously depict mural thickening, edema, restricted diffusion, mesenteric inflammatory changes, and complications such as strictures and fistulas [65–67]. However, standard protocols are often time-consuming and frequently include intravenous gadolinium-based contrast administration, which can reduce their feasibility for repeated examination and adds procedural complexity [68, 69]. These constraints are particularly relevant for patients requiring lifelong monitoring and for centers where scanner time is a limited resource [37, 70–72].

A strength of the present study is its large sample size and broad case-mix, which reflects real-world referral patterns rather than a narrowly selected cohort. The inclusion of pediatric patients is clinically meaningful because younger individuals may undergo repeated imaging over many years, and minimizing scan time may improve tolerability and reduce motion artifacts [73]. Similarly, postoperative patients represent a frequent and clinically important subgroup in which altered anatomy and variable motility can complicate interpretation; demonstrating robust agreement in a cohort that includes such patients supports applicability to routine clinical practice.

The substantial agreement observed for patient-level overall activity indicates that the abbreviated dataset can capture clinically meaningful inflammation when operational definitions are applied consistently. Agreement was also strong for bowel wall thickening, mural edema, and the comb sign—features that commonly drive clinical decisions and that align with a transmural conception of active disease. These findings reinforce the central premise of abbreviated protocols: high-resolution T2-weighted imaging combined with diffusion-weighted imaging can preserve much of the diagnostic information required for follow-up assessment of inflammatory activity.

In our analysis, the diagnostic performance of the abbreviated dataset for detecting overall active disease was high when full dataset interpretation was used as the reference standard. This choice of reference is appropriate for an imaging reliability study, because the objective is to determine whether an abbreviated acquisition can reproduce decisions derived from the established full protocol. At the same time, a full protocol interpretation is not an independent gold standard for histologic inflammation. The availability of correlations with biomarkers and endoscopy provides important context, suggesting that the abbreviated dataset findings track clinically relevant activity similarly to the full dataset, although prospective validation against independent endpoints remains desirable.

Complication assessment is a key consideration when adopting abbreviated protocols. In this study, agreement for fistulas, strictures, and abscesses ranged from moderate to substantial, indicating that clinically meaningful complications are frequently identifiable without contrast-enhanced sequences. A clinically relevant nuance was the slightly reduced conspicuity of very small abscesses on the abbreviated dataset. This limitation highlights a practical implementation message: abbreviated non-contrast protocols may be most appropriate for follow-up imaging in stable patients or for routine activity assessment, whereas full protocols should remain available for complex cases, suspected penetrating disease, severe systemic symptoms, or when excluding subtle collections is a primary clinical question.

The potential time savings associated with abbreviated protocols are operationally meaningful. Shorter examinations may improve patient compliance, reduce motion, and increase scanner throughput. Because the abbreviated dataset was derived by masking sequences from the full examination rather than acquired as a standalone scan, the abbreviated acquisition time represents an estimate based on included sequence times rather than a directly measured scan duration. Nonetheless, this estimate is directly relevant for protocol planning and provides an actionable representation of how abbreviated protocols could be implemented in routine scheduling, particularly in high-volume or resource-constrained settings.

Despite these limitations, the present study provides a comprehensive evaluation of an abbreviated non-contrast approach, integrating patient-level activity classification, feature-level agreement, complication assessment, image quality, acquisition time, reproducibility, and correlations with biomarkers and endoscopy. These multiple dimensions align with real clinical needs and provide a balanced picture of the potential benefits and limitations of adopting abbreviated protocols.

## Conclusions

An abbreviated non-contrast MRE protocol derived from a standard full protocol demonstrates substantial agreement with the full protocol for the patient-level assessment of Crohn’s disease activity and strong concordance for key inflammatory imaging features. This approach preserves diagnostic image quality and offers a substantial reduction in acquisition time, supporting its use for follow-up imaging in selected clinical scenarios where minimizing scan duration and avoiding intravenous contrast are priorities. Full contrast-enhanced protocols should remain available for complex cases and when subtle complications, such as very small abscesses, are strongly suspected.
